# Analysis of the Bovine Monocyte-Derived Macrophage Response to *Mycobacterium avium* Subspecies *Paratuberculosis* Infection Using RNA-seq

**DOI:** 10.3389/fimmu.2015.00023

**Published:** 2015-02-04

**Authors:** Maura E. Casey, Kieran G. Meade, Nicolas C. Nalpas, Maria Taraktsoglou, John A. Browne, Kate E. Killick, Stephen D. E. Park, Eamonn Gormley, Karsten Hokamp, David A. Magee, David E. MacHugh

**Affiliations:** ^1^Animal Genomics Laboratory, UCD School of Agriculture and Food Science, University College Dublin, Dublin, Ireland; ^2^Animal and Bioscience Research Department, Animal and Grassland Research and Innovation Centre, Teagasc, Dunsany, Ireland; ^3^Biological Agents Unit, Health and Safety Executive, Leeds, UK; ^4^Systems Biology Ireland, UCD Conway Institute of Biomolecular and Biomedical Research, University College Dublin, Dublin, Ireland; ^5^Tuberculosis Diagnostics and Immunology Research Centre, UCD School of Veterinary Medicine, University College Dublin, Dublin, Ireland; ^6^Smurfit Institute of Genetics, Trinity College Dublin, Dublin, Ireland; ^7^UCD Conway Institute of Biomolecular and Biomedical Research, University College Dublin, Dublin, Ireland

**Keywords:** cattle, immune response, Johne’s disease, macrophage, microarray, *Mycobacterium avium* subspecies *paratuberculosis*, RNA-sequencing, transcriptome

## Abstract

Johne’s disease, caused by infection with *Mycobacterium avium* subsp. *paratuberculosis*, (MAP), is a chronic intestinal disease of ruminants with serious economic consequences for cattle production in the United States and elsewhere. During infection, MAP bacilli are phagocytosed and subvert host macrophage processes, resulting in subclinical infections that can lead to immunopathology and dissemination of disease. Analysis of the host macrophage transcriptome during infection can therefore shed light on the molecular mechanisms and host-pathogen interplay associated with Johne’s disease. Here, we describe results of an *in vitro* study of the bovine monocyte-derived macrophage (MDM) transcriptome response during MAP infection using RNA-seq. MDM were obtained from seven age- and sex-matched Holstein-Friesian cattle and were infected with MAP across a 6-h infection time course with non-infected controls. We observed 245 and 574 differentially expressed (DE) genes in MAP-infected versus non-infected control samples (adjusted *P* value ≤0.05) at 2 and 6 h post-infection, respectively. Functional analyses of these DE genes, including biological pathway enrichment, highlighted potential functional roles for genes that have not been previously described in the host response to infection with MAP bacilli. In addition, differential expression of pro- and anti-inflammatory cytokine genes, such as those associated with the IL-10 signaling pathway, and other immune-related genes that encode proteins involved in the bovine macrophage response to MAP infection emphasize the balance between protective host immunity and bacilli survival and proliferation. Systematic comparisons of RNA-seq gene expression results with Affymetrix^®^ microarray data generated from the same experimental samples also demonstrated that RNA-seq represents a superior technology for studying host transcriptional responses to intracellular infection.

## Introduction

Johne’s disease, caused by infection with *Mycobacterium avium* subsp. *paratuberculosis* (MAP) is a chronic granulomatous enteritis of ruminants, both domestic and wild, including cattle, sheep, deer, and other mammalian species (1). Furthermore, there is some evidence, albeit contentious, suggesting that infection with MAP may be associated with Crohn’s disease in humans (2–4). While prevalence figures of Johne’s disease in cattle are difficult to determine – due, in part, to limited sensitivity and specificity of MAP diagnostic tests – current estimates in European countries vary from 31 to 71% (5–8). In the United States, Johne’s disease is estimated to cost the economy between $200 million and $1.5 billion annually, with that figure rising concurrently with herd-level MAP prevalence (9, 10).

The primary route of MAP transmission is believed to be fecal-oral or through ingestion of infected colostrum (11, 12). Once internalized, infectious bacilli cross the intestinal mucosa by penetrating specialized microfold cells (M cells) or enterocytes, which are located in the epithelium lining of the dome areas of Peyer’s patches (13–15). The bacilli then traverse the M cells by transcytosis and migrate to the basolateral side of the cell where they are recognized and phagocytosed by intestinal macrophages. Macrophage recognition of MAP bacilli is mediated by host pathogen recognition receptors (PRRs), including cell-surface Toll-like receptors (TLRs) and intracellular NOD-like receptors (NLRs) (16, 17); indeed, it has been demonstrated that TLR2, TLR4, and NOD2 can independently recognize MAP cellular components (18). Infected macrophages secrete pro-inflammatory cytokines, such as IL-1B and TNF, which activate an early protective T_H_1 response characterized by the release of IFN-γ from T-cells. IFN-γ activates the antimicrobial mechanisms of the macrophage that destroys the internalized pathogen and also induces the development of granulomas that actively contain infection in the majority of animals such that clinical signs do not usually manifest (19–21).

The outcome of MAP infection is dependent on the interaction between infected macrophages and T-cells; progression to clinical infection is thought to develop in animals that fail to eradicate the pathogen with a concomitant shift in the immune system from a protective cellular response to a non-protective humoral response. Consequently, both humoral and cellular immune responses can exist simultaneously in infected individuals and it is possible for MAP bacilli to latently infect animals by persisting in host macrophages for prolonged periods and later become reactivated if, for example, the animal subsequently becomes immunosuppressed (22). MAP has the capacity to survive and subvert the macrophage response to ensure its survival and replication (20, 21, 23, 24). In general, the interactions between the macrophage and MAP upon infection are comparable to those observed for other pathogenic mycobacteria such as *M. tuberculosis* and *M. bovis* (22). In this context, MAP prevents phagosome maturation, thus facilitating bacterial survival in phagosomes, which in turn provide a niche for further bacterial growth (25). The mechanisms used by MAP to do this are complex but primarily involve the modulation of various cell signaling pathways through interaction with cell membrane receptors, inhibiting phagosome acidification and phagolysosome fusion, and reducing antigen presentation to the immune system (26). MAP, in common with other mycobacterial pathogens also subverts cell death processes, particularly apoptosis to inhibit antigen presentation and the subsequent development of an effective immune response (25). It has also been suggested that inhibition of apoptosis may contribute to the large numbers of infected macrophages that persist in affected tissues (10, 25). Persistence of MAP in macrophages underlies the progression to clinical disease, which is characterized by immunopathology, proliferation of the pathogen, dissemination infection through the host, and ultimately fecal shedding of the pathogen from the host, thus maintaining the cycle of infection (11, 12, 27).

Through modulation and subversion of the bovine host macrophage, MAP promotes its short- and long-term survival. Therefore, analysis of the macrophage transcriptome in response to MAP infection can shed light on the cellular processes underlying pathogen–macrophage interactions and how the perturbation of these pathways is associated with the pathogenesis of Johne’s disease. In recent years, RNA sequencing (RNA-seq) has provided unprecedented opportunities for gene expression analysis of host response to infection, including unbiased whole-transcriptome profiling, sense and antisense transcription analysis, the characterization of new classes of RNA, and the identification of novel mRNA splice variants (28, 29).

Previously, we used the Affymetrix^®^ GeneChip^®^ Bovine Genome Array to study host gene expression in RNA extracted from MAP-infected and non-infected control bovine monocyte-derived macrophages (MDM) across a 24 h time course (30). Our analysis revealed a marked reduction in the number of differentially expressed (DE) genes at the 24 h time point compared to the two earlier infection time points; indeed, these results indicated that majority of transcriptional changes induced by infection occur within the first 6 h of infection, with differential gene expression having largely abated 24 h post-infection (hpi). Consequently, for the present study, we describe analysis of the same RNA samples from the 2 and 6 hpi time points using RNA-seq to enhance detection of host macrophage mRNA transcripts and molecular pathways perturbed and modulated by MAP infection.

## Materials and Methods

### Ethics statement

All animal procedures were carried out according to the provisions of the Cruelty to Animals Act (Irish Department of Health and Children license number B100/3939) and ethical approval for the study was obtained from the UCD Animal Ethics Committee (protocol number AREC-P-07-25-MacHugh).

### Animals

Seven age-matched (4-year old) Holstein-Friesian females were used for this study and have previously been described by our group. These animals had been maintained under uniform housing conditions and nutritional regimens at the UCD Lyons Research Farm (Newcastle, County Kildare, Ireland). The animals did not have a recent history of Johne’s disease and were also negative for infection with *M. bovis* (30).

### MDM preparation and infection and RNA purification

The methods used to isolate, purify, and infect bovine MDM with MAP have been previously described by our group (29–32). MDM from seven age-matched, female Holstein-Friesian cattle were infected *in vitro* with a clinical isolate of MAP [multiplicity of infection (MOI) of 2 bacilli:1 MDM] and parallel non-infected control MDM samples were also generated.

Total RNA was extracted from each individual sample and purified individually at 0, 2, and 6 hpi and used to prepare pooled strand-specific RNA-seq libraries as previously described by us (29, 33). RNA was extracted using an RNeasy kit incorporating an on-column DNase treatment step (Qiagen Ltd., Crawley, UK) according to the manufacturer’s instructions. The quantity and quality of the RNA was assessed using a NanoDrop™ 1000 spectrophotometer (Thermo Fisher Scientific, Waltham, MA, USA) and an Agilent 2100 Bioanalyzer with an RNA 6000 Nano LabChip kit (Agilent Technologies Ltd., Cork, Ireland). A260/280 ratios >2.0 and RNA integrity numbers (RINs) >8.5 were obtained for all total RNA samples purified across the infection time course.

### Strand-specific RNA-seq library preparation

The protocol used for RNA-seq library preparation was adapted from a protocol previously published by our group (29). Thirty-five strand-specific Illumina^®^ RNA-seq libraries were generated (seven libraries for the MAP-infected and control groups at the 2 and 6 hpi time points and seven 0 h time point control samples) using 150–200 ng of total RNA. Samples were heated at 65°C for 5 min to disrupt RNA secondary structure and purification of poly(A)+ RNA was performed using the Dynabeads^®^ mRNA DIRECT™ Micro Kit according to the manufacturer’s instructions (Invitrogen™/Life Technologies Ltd., Paisley, UK). Purified poly(A)+ RNA was then fragmented using 1 × RNA Fragmentation Reagent (Ambion^®^/Life Technologies Ltd., Warrington, UK) for 5 min at 70°C and precipitated using 68 mM sodium acetate pH 5.2 (Ambion^®^), 227 ng/μl glycogen (Ambion^®^) and 30 μl of 100% ethanol (Sigma-Aldrich Ltd., Dublin, Ireland). The RNA pellets obtained were then washed with 80% ethanol, air-dried for 10 min at room temperature and then re-suspended in 10.5 μl of DNase- and RNase-free molecular biology-grade H_2_O.

Synthesis of first strand cDNA was performed by incubating fragmented RNA with 261 mM Random Hexamer Primers (Invitrogen™), 1× first strand buffer (Invitrogen™); 10 mM DTT (Invitrogen™); 0.5 mM dNTPs; 20 U RNaseOUT™ Recombinant Ribonuclease Inhibitor; and 200 U SuperScript^®^ II Reverse Transcriptase (Invitrogen™) using the following temperature profile: 25°C for 10 min, 42°C for 50 min, and 70°C for 15 min. First strand synthesis reaction mixtures were then purified using MicroSpin™ G-50 columns according to the manufacturer’s instructions (GE Healthcare UK Ltd., Buckinghamshire, UK).

Second strand cDNA synthesis, involving the incorporation of uracil, was performed by adding the first strand cDNA synthesis reaction to a second strand reaction mix consisting of 0.065× first strand buffer (Invitrogen™); 1× second strand buffer (Invitrogen™); a dNTP solution consisting of a final concentration of 0.3 mM dATP, dCTP, dGTP (Sigma-Aldrich), and 0.3 mM dUTP (Bioline Reagents Ltd., London, UK); 1 mM DTT (Invitrogen™); 2 U RNase H (Invitrogen™); and 50 U *E. coli* DNA Polymerase I (Invitrogen™). Reactions were incubated at 16°C for 2.5 h. The double stranded cDNA was subsequently purified using a QIAquick PCR Purification kit (Qiagen) according to the manufacturer’s instructions and eluted in 30 μl of the provided elution buffer.

Blunt-end repair of cDNA samples was performed in 100 μl reaction volumes containing 1× T4 DNA ligase buffer with 10 mM dATP (New England Biolabs^®^ Inc., Ipswich, MA, USA), 0.4 mM of each dNTP (Invitrogen™), 15 U T4 DNA polymerase (New England Biolabs^®^), 5 U DNA Polymerase I Large (Klenow) Fragment (New England Biolabs^®^), and 50 U T4 polynucleotide kinase (New England Biolabs^®^). Reactions were incubated at 20°C for 30 min and the cDNA was then purified using a QIAquick PCR Purification Kit (Qiagen) according to the manufacturer’s instructions and eluted in 32 μl of the provided elution buffer.

Illumina^®^ RNA-seq adaptor ligation reactions (50 μl volumes) were performed using 21 μl of each of the phosphorylated blunt-ended cDNA (with 3′-dATP overhangs) samples and 1× Quick DNA ligase buffer (New England Biolabs^®^); 30 nM custom indexed single-read adaptors (Table S1 in Supplementary Material) and 15 U T4 DNA ligase (Invitrogen™). Reaction mixes were incubated at room temperature for 15 min and purified using a QIAquick MinElute Kit according to the manufacturer’s instructions (Qiagen) and eluted in 10 μl of the provided elution buffer. Adaptor-ligated cDNA was gel-purified using 2.5% agarose gels stained with 1 μg/ml ethidium bromide (Invitrogen™). Gels were electrophoresed at 100 V using 1× TAE buffer (Invitrogen™) for 75 min at room temperature. Size-fractionated bands corresponding to 200 bp (+50 bp) were excised from each sample and purified using a QIAquick Gel Extraction kit (Qiagen) according to the manufacturer’s instructions and eluted in 30 μl of elution buffer. For generation of strand-specific RNA-seq libraries, the second strand of the gel-purified adapter-ligated cDNA containing uracil was enzymatically digested in 30 μl reaction volumes containing 1× Uracil-DNA Glycosylase buffer and 1 U Uracil-DNA Glycosylase (Bioline). These reactions were incubated at 37°C for 15 min followed by 94°C for 10 min.

PCR enrichment amplifications (25 μl) containing 9 μl of second strand-digested, adaptor-ligated cDNA; 1× Phusion^®^ High-Fidelity DNA polymerase buffer (New England Biolabs); 334 nM each Illumina^®^ PCR primer (Illumina^®^ Inc., San Diego, CA, USA); 0.4 mM each of dATP, dCTP, DGTP, and dTTP (Invitrogen™); and 1 U Phusion^®^ High-Fidelity DNA polymerase (New England Biolabs^®^). PCR amplification reactions were performed with the following temperature cycling profile: 98°C initial denaturation for 30 s; 18 cycles of 98°C for 10 s, 65°C for 30 s, and 72°C for 30 s; and 72°C final extension step for 5 min. PCR products were visualized following electrophoresis on a 2% agarose gel stained with ethidium bromide (0.6 μg/ml; Invitrogen™) and purified to remove PCR-generated adaptor-dimers using an Agencourt AMPure XP kit (Beckman Colter Genomics, Danvers, MA, USA) according to the manufacturer’s instructions with final elution in 30 μl of 1× TE buffer.

All RNA-seq libraries were quantified using a Qubit^®^ Fluorometer (Invitrogen™). RNA-seq library quality was assessed using an Agilent Bioanalyzer and Agilent High sensitivity DNA chip (Agilent) and confirmed that insert sizes were 200–250 bp for all individual libraries. Individual RNA-seq libraries were standardized and pooled in equimolar quantities (10 μM for each individual library). The quantity and quality of the final pooled library was assessed as described above prior to sequencing.

Cluster generation and sequencing of the pooled RNA-seq libraries was performed on an Illumina^®^ HiSeq 2000 sequencer according to the manufacturer’s instructions. These RNA-seq data have been deposited in the NCBI Gene Expression Omnibus (GEO) database with experiment series accession number GSE62048.

### Bioinformatics and statistical analysis of RNA-seq data

All of the bioinformatics pipeline information and associated scripts used for computational analyses are available in a GitHub repository at https://github.com/mauracasey/RNA-sequencing. These analyses were performed on a 32-node Compute Server running Linux Ubuntu (version 12.04.2) with 256 GB of RAM and 24 TB of hard disk drive storage.

Initial quality checks were performed on each of the raw reads data files using the FastQC software (version 0.10.1)^1^ to determine the most appropriate read quality filtering methodology. Consequently, a custom perl script was used to deconvolute sequence reads obtained from the flow cell into 35 individual libraries using the indexed barcoded adapters (the script was optimized to work with single-end reads and a six nucleotide barcode at the 5′-end of each read).

For initial sequence adapter removal and quality filtering, appropriate parameters were used with the custom perl script to filter out reads containing adapter sequence (allowing up to three mismatches) and reads below a sequence quality threshold (discard reads with more than 25% bases with a phred score <20); all reads were also trimmed of 20 nucleotides at the 3′-ends.

The FastQC package was used to further assess the filtered individual fastq files, revealing that no further filtering steps were required. The STAR RNA-seq aligner software package (version 2.3.0) (34) was used to align filtered sequence reads to the most recent version of the *Bos taurus* reference genome [UMD3.1.73; (35)]. Aligned sequence reads in individual SAM files were then used for a final FastQC quality check step to detect quality score biases in the aligned reads and all samples successfully passed.

The featureCounts tool, which is part of Subread software package (36, 37), was used to perform count summarization of sense genes. Reads were assigned to a gene if they were not multi-hit reads and if the mapped location was associated with a unique gene on the sense strand. Differential gene expression analysis was performed using the Bioconductor edgeR package (38) with the gene raw counts obtained from featureCounts. The BioMart tool was used first for gene annotation with Ensembl gene IDs (39). Ribosomal RNA genes were filtered out and lowly expressed genes were also removed with a minimally set threshold of one count per million (CPM) in at least seven individual libraries (the choice of seven libraries is based on the sample size of each treatment group) (38). For each library, a normalization factor was calculated based on RNA composition among libraries (computed using trimmed mean of *M*-values). For the present study, at this stage the seven 0 h control samples were removed from the data set and not used for any subsequent bioinformatics, differential gene expression, or downstream data analyses.

Using the edgeR package (38), DE genes between MAP-infected versus non-infected control MDM samples for each time point post-infection (2 and 6 hpi) were obtained using paired-sample statistics by fitting a negative binomial generalized linear model to each gene. Multiple-testing correction was performed using the Benjamini–Hochberg method (40) with a false discovery rate (FDR)-adjusted threshold of ≤0.05.

### Functional analyses of DE genes obtained using RNA-seq

The RNA-seq DE gene lists obtained for each time point post-infection were used for downstream systems analysis to identify important cellular pathways with the Ingenuity^®^ Systems Pathway Analysis Knowledgebase (IPA^2^;Summer Release, June 2014). This approach was used to identify canonical pathways that were overrepresented based on the list of DE genes at each of the two time points post-infection using Fisher’s exact test (FDR-adjusted *P* value threshold ≤0.05).

The GOseq Bioconductor package (41) was used to determine gene ontology (GO) biological process functions that were enriched based on the RNA-seq DE gene lists obtained for each time point post-infection (Bonferroni-adjusted *P* value threshold ≤0.05).

### Comparative analysis of microarray data

The raw microarray data generated from the 35 total RNA samples used for the RNA-seq DE gene and downstream analyses (MDM from seven animals at 0 h, 2, and 6 hpi with the corresponding control samples) were retrieved from the NCBI GEO repository (42) with the accession number GSE35185 (30). The Affymetrix^®^ GeneChip^®^ Bovine Genome Array used to generate these data contains 24,072 probe sets representing more than 23,000 gene transcripts. The retrieved microarray data were then analyzed with a number of different Bioconductor packages (43) using the UMD3.1.73 build of the bovine genome (35). The Factor Analysis for Robust Microarray Summarization (FARMS) algorithm was used to normalize the microarray data (44) and these normalized data were then filtered for informative probes sets using the FARMS informative/non-informative (I/NI) calls unsupervised feature selection method (45).

To compare data generated using the two different gene expression technologies, microarray probe sets were annotated with bovine Ensembl gene IDs from the *B. taurus* reference genome build used to annotate the RNA-seq data [UMD3.1.75; (35)] using the Bioconductor biomaRt package (39). DE genes were detected between experimental groups using the Linear Models for Microarray Data (LIMMA) Bioconductor package (46). A Benjamini–Hochberg multiple-testing correction of *P* ≤ 0.05 was used for all DE genes (40) and the Euclidean distance was used as the distance metric for MDS plotting.

## Results

### Preliminary analysis and summary statistics for RNA-seq data

The 35 RNA-seq libraries used for the present study were sequenced across six lanes of an Illumina^®^ HiSeq 2000 sequencing apparatus and generated mean values per library of 26.72 million raw reads, of which 20.02 million reads (74.94%) remained after adapter sequence and poor quality reads filtering (Figure S1A in Supplementary Material). Alignment of the filtered RNA-seq reads to the *B. taurus* reference genome (UMD3.1.73) yielded mean values per library of 16.19 million reads (80.85%) mapping to unique locations in the bovine genome, 1.66 million reads (8.31%) mapping to multiple locations in the genome, and 2.17 million reads (10.84%) not mapping to any genome location (Figure S1B in Supplementary Material). Further analysis, focusing on the uniquely mapping reads demonstrated that a mean of 11.91 million reads (73.60%) per library were assigned to annotated sense regions of the genome. Only these sequence reads were then used to calculate raw counts per sense gene and for downstream differential gene expression and systems biology analyses. In addition, a mean value per library of 4.27 million reads (26.40%) could not be assigned to annotated genome locations or were assigned to overlapping annotated genomic regions (Figure S1C in Supplementary Material). The detailed number of reads per individual RNA-seq library at each stage of the analysis is provided in Table S1 in Supplementary Material.

Analysis of the gene coverage based solely on sense sequence information, demonstrated that of the 24,616 *B. taurus* genes annotated in Ensembl (release 73), 17,571 of these genes (71.4%) had at least one sequence read count (i.e., one mapped read) in at least one of the 35 individual RNA-seq libraries. These 17,571 genes were further filtered by removing lowly expressed genes, yielding 11,813 sense-strand genes (48% of annotated *B. taurus* genes) that were considered for downstream analyses.

### Analysis of differential gene expression from RNA-seq data

Following preliminary RNA-seq analysis, the sequence reads that mapped to unique locations in the *B. taurus* reference genome were used to generate lists of DE genes between the MAP-infected and control MDM groups at 2 and 6 hpi (the 0 h control MDM samples were not used for this phase of the analysis). Using an FDR threshold of ≤0.05, at 2 hpi 209 genes were significantly upregulated and 36 genes were significantly downregulated (Table S2 in Supplementary Material). It is important to note that the number of DE genes observed between MAP-infected and control MDM samples at 2 hpi was markedly higher for upregulated genes (209) compared to the downregulated genes (36). Inspection of the list of DE genes in Table S2 in Supplementary Material at the 2 hpi time point reveals that many of the top-ranked DE genes by FDR-adjusted *P* value have immune-related functions; for example, the v-maf avian musculoaponeurotic fibrosarcoma oncogene homolog F gene (*MAFF*); the nuclear factor of kappa light polypeptide gene enhancer in B-cells inhibitor, delta gene (*NFKBID*), the chemokine (C–C motif) ligand 3 gene (*CCL3*), and the chemokine (C–C motif) ligand 4 gene (*CCL4*). Table 1 shows the top 10 upregulated and top 10 downregulated DE genes between MAP-infected and control MDM samples at 2 hpi ranked by fold-change and with FDR-adjusted *P* values ≤0.05.

**Table 1:** **The top 10 upregulated and downregulated DE genes (FDR ≤ 0.05) for MAP-infected versus control MDM samples at 2 hpi as ranked by fold-change**.

Gene symbol	Ensembl ID	Gene name	Log_2_ fold-change	*P* value	FDR-adjusted *P* value
*CSF3*	ENSBTAG00000021462	Colony stimulating factor 3 (granulocyte)	+8.05	0.000000	0.000002
*CXCL3*	ENSBTAG00000037778	Chemokine (C–X–C motif) ligand 3	+6.24	0.000000	0.000000
*TNFAIP6*	ENSBTAG00000007239	Tumor necrosis factor, alpha-induced protein 6	+6.07	0.000001	0.000144
*CCL20*	ENSBTAG00000021326	Chemokine (C–C motif) ligand 20	+5.95	0.000001	0.000133
*IL1B*	ENSBTAG00000001321	Interleukin 1, beta	+5.64	0.000000	0.000000
*TNFSF9*	ENSBTAG00000046266	Tumor necrosis factor (ligand) superfamily, member 9	+5.53	0.000000	0.000000
*RND1*	ENSBTAG00000018773	Rho family GTPase 1	+5.50	0.000000	0.000000
*PTX3*	ENSBTAG00000009012	Pentraxin 3, long	+5.35	0.000000	0.000000
*CXCL2*	ENSBTAG00000027513	Chemokine (C–X–C motif) ligand 3	+5.11	0.000000	0.000059
*TNF*	ENSBTAG00000025471	Tumor necrosis factor	+5.03	0.000000	0.000000
–	ENSBTAG00000048135	Uncharacterized	−3.86	0.000881	0.043735
*RAB3A*	ENSBTAG00000010635	RAB3A, member RAS oncogene family	−2.10	0.000699	0.036718
*OSM*	ENSBTAG00000016163	Oncostatin M	−1.91	0.000037	0.003243
*FOS*	ENSBTAG00000004322	FBJ murine osteosarcoma viral oncogene homolog	−1.89	0.000000	0.000034
*POU3F1*	ENSBTAG00000012061	POU class 3 homeobox 1	−1.88	0.000987	0.048195
*ANKRD63*	ENSBTAG00000046052	Ankyrin repeat domain 63	−1.38	0.000114	0.008605
*SMAD6*	ENSBTAG00000000625	SMAD family member 6	−1.36	0.000001	0.000131
*PIK3IP1*	ENSBTAG00000010667	Phosphoinositide-3-kinase interacting protein 1	−1.31	0.000000	0.000013
*PDK4*	ENSBTAG00000014069	Pyruvate dehydrogenase kinase, isozyme 4	−1.19	0.000167	0.011389
*DUSP7*	ENSBTAG00000021912	Dual specificity phosphatase 7	−1.11	0.000059	0.004862

Notably, the difference between the numbers of upregulated and downregulated DE genes (FDR ≤ 0.05) was not as marked at the 6 hpi time point (342 upregulated versus 232 downregulated genes). These results are in broad agreement with the previous microarray analysis (590 upregulated genes and 384 downregulated genes with an FDR-adjusted *P* value ≤0.10) (30). Top ranking genes by FDR-adjusted *P* value for 6 hpi (Table S2 in Supplementary Material) included the mucosal vascular address in cell adhesion molecule 1 gene (*MADCAM1*), the family with sequence similarity 129, member A gene (*FAM129A*), the CD40 molecule, TNF receptor superfamily member 5 gene (*CD40*), and the phospholipid transfer protein gene (*PLTP*). Table 2 shows the top 10 upregulated and top 10 downregulated DE genes between MAP-infected and control MDM samples at 6 hpi ranked by fold-change and with FDR-adjusted *P* values ≤0.05.

**Table 2:** **The top 10 upregulated and downregulated DE genes (FDR ≤ 0.05) for MAP-infected versus control MDM samples at 6 hpi as ranked by fold-change**.

Gene symbol	Ensembl ID	Gene name	Log_2_ fold-change	*P* value	FDR-adjusted *P* value
*–*	ENSBTAG00000046848	Uncharacterized	+7.50	0.000145	0.006032
*LOXL4*	ENSBTAG00000020895	Lysyl oxidase-like 4	+5.42	0.000000	0.000005
*GJB2*	ENSBTAG00000017425	Gap junction protein, beta 2, 26 kDa	+4.83	0.000001	0.000097
*FFAR4*	ENSBTAG00000000437	Free fatty acid receptor 4	+4.58	0.000275	0.009924
*–*	ENSBTAG00000013711	Uncharacterized	+4.50	0.000012	0.000839
*STOML3*	ENSBTAG00000018232	Stomatin-like protein 3	+4.46	0.000003	0.000258
*SAA3*	ENSBTAG00000022396	Serum amyloid A 3	+4.31	0.000000	0.000000
*AQPEP*	ENSBTAG00000016644	Laeverin	+4.19	0.000028	0.001678
*CD38*	ENSBTAG00000013569	CD38 molecule	+4.16	0.000002	0.000168
*M-SAA3.2*	ENSBTAG00000010433	mammary serum amyloid A3.2	+4.04	0.000000	0.000011
*OPRD1*	ENSBTAG00000003202	Opioid receptor, delta 1	−3.21	0.001890	0.041982
*TPBGL*	ENSBTAG00000019622	Trophoblast glycoprotein-like	−3.16	0.000080	0.003833
*CCDC30*	ENSBTAG00000004585	Coiled-coil domain containing 30	−2.70	0.000111	0.005004
*TNFSF18*	ENSBTAG00000047412	Tumor necrosis factor (ligand) superfamily, member 18	−2.35	0.000063	0.003196
*KIT*	ENSBTAG00000002699	v-kit Hardy-Zuckerman 4 feline sarcoma viral oncogene homolog	−2.21	0.000305	0.010742
*SLC7A8*	ENSBTAG00000007415	Solute carrier family 7, member 8	−2.19	0.000001	0.000083
*STON2*	ENSBTAG00000025308	Stonin 2	−2.08	0.000500	0.016302
*ARHGAP26*	ENSBTAG00000027151	Rho GTPase activating protein 26	−1.91	0.000000	0.000000
*SLCO2B1*	ENSBTAG00000015596	Solute carrier organic anion transporter family, member 2B1	−1.85	0.000002	0.000171
*–*	ENSBTAG00000001476	Uncharacterized	−1.81	0.000431	0.014268

**Figure 1:**
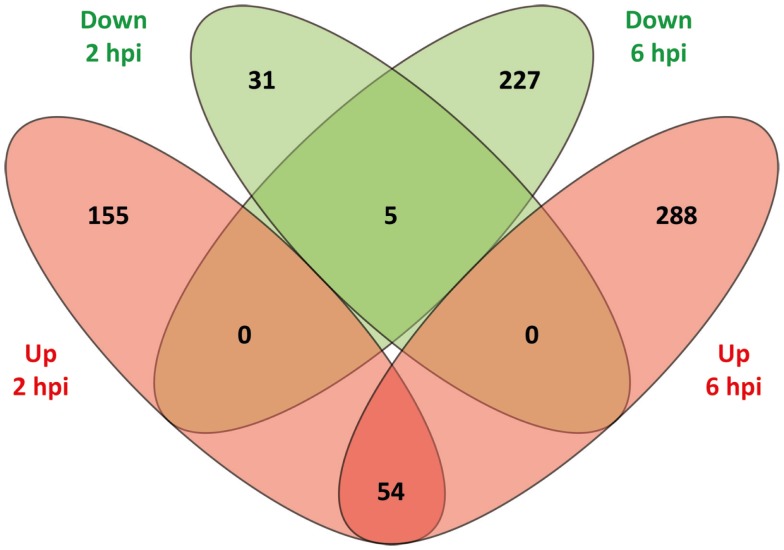
**A Venn diagram showing the numbers of DE genes identified at 2 and 6 hpi**. Overlap comparison of DE genes detected in MAP-infected MDM versus control non-infected MDM between 2 and 6 hpi using the RNA-seq dataset. Sets of upregulated genes are represented in red and sets of downregulated genes are shown in green.

The DE genes were compared according to direction of expression between 2 and 6 hpi. As shown in Figure 1, 59 genes (54 upregulated and 5 downregulated) were DE at both time points while also displaying the same direction of expression. By comparison, 186 genes (155 upregulated and 31 downregulated) and 515 genes (288 upregulated and 227 downregulated) were observed to be uniquely DE at 2 and 6 hpi, respectively. The relatively low overlap of DE genes between the two time points most likely represents evolution of the MDM transcriptional response to MAP infection over the time course.

### Functional categorization of DE genes detected with RNA-seq

**Figure 2:**
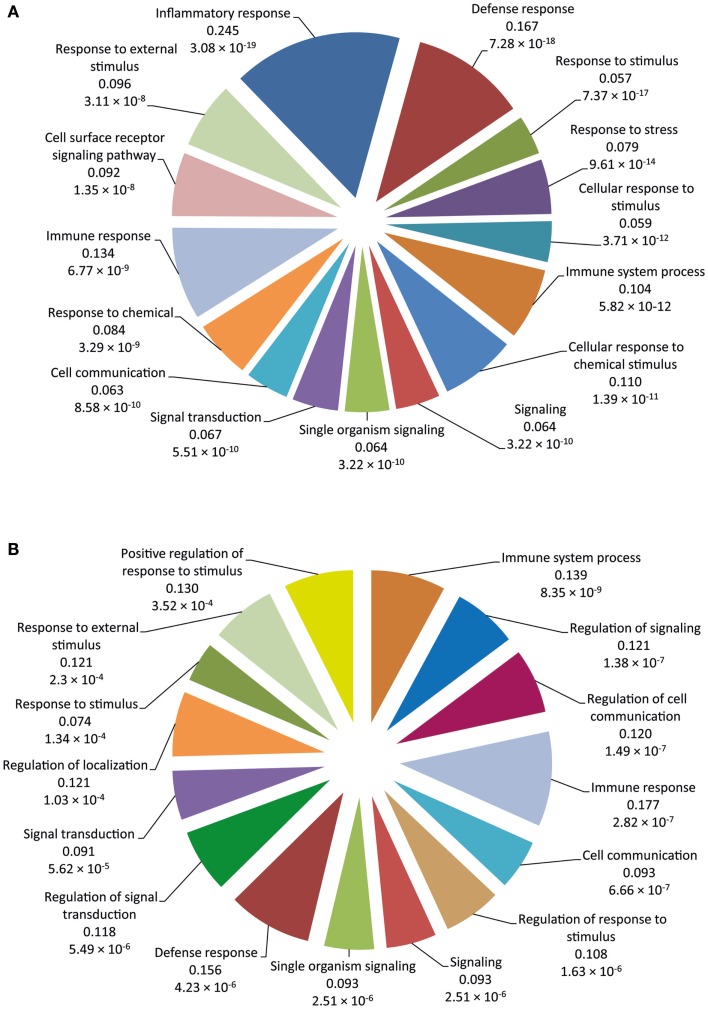
**The 15 top-ranked overrepresented biological process GO functions identified using the GOseq package**. **(A)** Pie chart of the enriched biological processes generated from DE genes at 2 hpi using the RNA-seq dataset. **(B)** Pie chart of the enriched biological processes generated from DE genes at 6 hpi using the RNA-seq dataset. The values below each function represent the ratio of DE genes versus the total gene set for each functional category and the Bonferroni-adjusted *P* value.

Functional categorization of DE genes was performed using the Bioconductor GOseq package (41) at 2 and 6 hpi time points to identify enriched Biological Process GO functions. At 2 hpi, we identified 149 significantly overrepresented Biological Processes (Bonferroni-adjusted *P* value ≤0.05) (Table S3 in Supplementary Material). Among the top ranked (based on Bonferroni-adjusted *P* values) Biological Processes were *inflammatory response*, *defense response*, *response to stimulus*, *response to stress*, *immune system process*, *signaling*, and *signal transduction* (Figure 2A). In addition, at 6 hpi, there were 40 significantly over-represented Biological Processes (Bonferroni-adjusted *P* value ≤0.05) (Table S4 in Supplementary Material), including *immune system process*, *regulation of signaling*, *regulation of cell communication*, *immune response*, *cell communication*, *regulation of response to stimulus*, *signaling*, and *defense response* (Figure 2B). The significantly overrepresented Biological Processes are relatively similar between the two post-infection time points and are, for the most part, associated with immunobiology.

**Figure 3:**
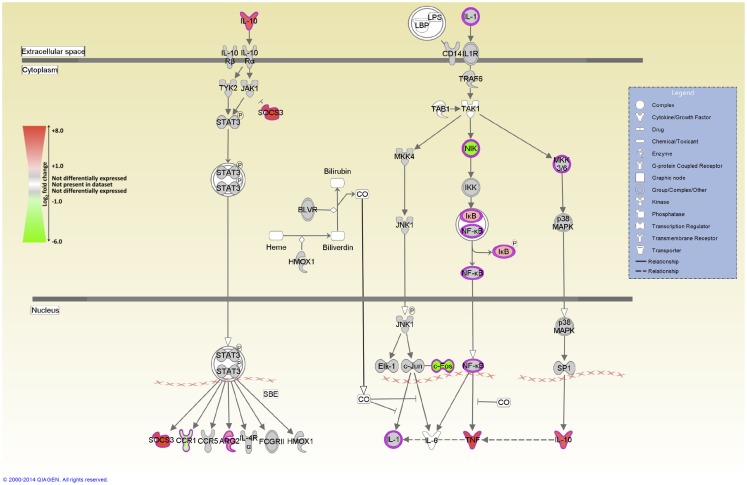
**The top-ranked enriched canonical pathway identified using IPA at 2 hpi – the *IL-10 signaling* pathway**. Red shading indicates increased expression in MAP-infected MDM relative to the non-infected control MDM. Green shading indicates decreased expression in MAP-infected MDM relative to the non-infected control MDM. White and gray shading indicates non-expression and non-differential expression, respectively.

**Figure 4:**
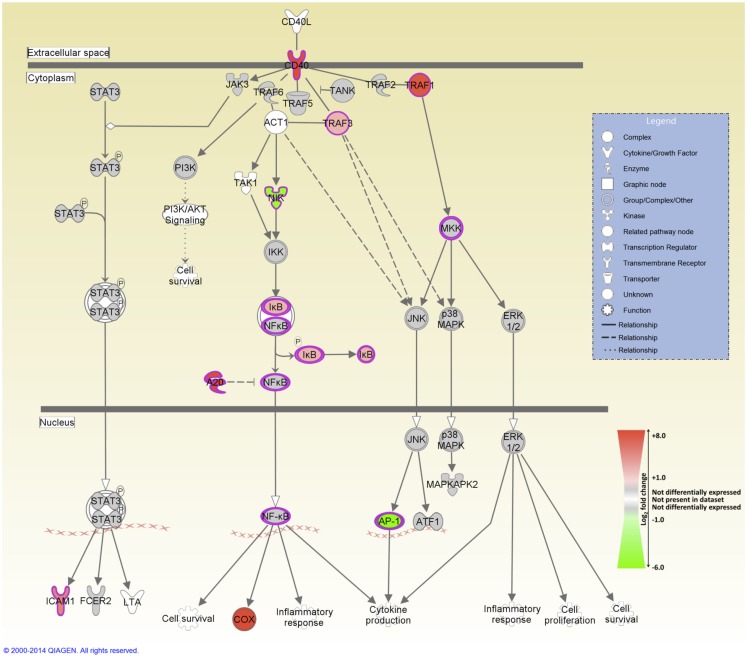
**The fourth-ranked enriched canonical pathway identified using IPA at 2 hpi – the *CD40 signaling* pathway**. Red shading indicates increased expression in MAP-infected MDM relative to the non-infected control MDM. Green shading indicates decreased expression in MAP-infected MDM relative to the non-infected control MDM. White and gray shading indicates non-expression and non-differential expression, respectively.

IPA was used to identify the canonical pathways that were enriched for DE genes at both post-infection time points. In the current study, we identified 155 and 177 canonical pathways that were significantly enriched (FDR-adjusted *P* value ≤0.05) at 2 and 6 hpi, respectively. It is notable that all of the top 10 ranking canonical pathways identified at 2 hpi have immunobiological functions (Table S5 in Supplementary Material). These canonical pathways include *IL-10 signaling*, the first ranked pathway, which is shown overlaid with gene expression results in Figure 3 and *CD40 signaling*, the fourth ranking pathway, which is presented in Figure 4. The top ranking canonical pathways at 6 hpi (Table S6 in Supplementary Material) included *Interferon signaling* (second ranked pathway), *IL-15 signaling* (third ranked pathway), and *P13K signaling in B lymphocytes* (fourth ranked pathway).

### Comparative analyses of DE genes detected using RNA-seq and microarray technologies

Total RNA samples purified from the MAP-infected and control non-infected MDM were analyzed previously by us using the Affymetrix^®^ Bovine Genome Array (30). To directly compare the gene expression results between the RNA-seq and microarray platforms, we re-analyzed the microarray data for the 0 h, 2, and 6 hpi time points (35 samples).

Of the 24,072 probe sets represented on the array, 11,259 probe sets were informative that represented 5,542 unique genes with Ensembl bovine gene ID. Prior to differential gene expression analysis, the data from the 11,259 informative probes was used to generate multi-dimensional scaling (MDS) plots at 2 and 6 hpi. Using the same procedure, MDS plots were also produced from the equivalent RNA-seq data at 2 and 6 hpi using all detectable genes (11,813 genes) (Figure S2 in Supplementary Material). Inspection of these MDS plots shows relatively small separation of individual samples according to their infection status by MDS dimension axis at either time point post-infection for the two technologies. This feature of both gene expression data sets may be due to the signal from DE genes being obscured by the background gene expression noise of the majority of detectable genes.

Further analysis of the microarray data showed that 315 genes (201 upregulated and 114 downregulated) were significantly DE at 2 hpi (FDR-adjusted *P* value ≤0.05). Comparison of overlapping DE genes between the microarray and RNA-seq data sets revealed 134 DE genes that displayed the same direction of expression with both technologies and 292 genes that were only DE on a single platform (181 DE genes unique to the microarray and 111 DE genes unique to RNA-seq) (Figure S3A in Supplementary Material).

At 6 hpi, 466 genes (307 upregulated and 159 downregulated) were DE in the MAP-infected relative to the control MDM based on the microarray data (FDR-adjusted *P* value ≤0.05). Comparison of common DE genes across the microarray and RNA-seq platforms revealed 189 DE genes displaying the same direction of expression for the two technologies. The remaining 662 genes were detected as DE using a single platform (277 DE genes unique to the microarray and 385 DE genes unique to RNA-seq) (Figure S3B in Supplementary Material).

Detailed information for all DE genes detected using the Affymetrix^®^ microarray in MAP-infected versus control non-infected MDM samples at 2 and 6 hpi is provided in Table S7 in Supplementary Material.

### Estimation of dynamic range from RNA-seq and microarray data

To estimate the dynamic range of the RNA-seq and microarray platforms, the log_2_ reads per kilobase per million mapped reads (RPKM) from the RNA-seq data and the log_2_ intensities from the microarray data were analyzed as described by Nalpas et al. (29). The lowest gene expression value was subtracted from the highest gene expression value for each platform. For the RNA-seq platform, a log_2_ dynamic range of 25.31 was estimated based on the *FAT3* gene (ENSBTAG00000004081, log_2_ RPKM = −9.15) and the *FTH1* gene (ENSBTAG00000011184, log_2_ RPKM = 16.16). For the microarray platform, this calculation yielded an estimated log_2_ dynamic range of 13.56 based on the *ZCCHC8* gene (ENSBTAG00000006114, log_2_ intensity = 2.03) and the *B2M* gene (ENSBTAG00000012330, log_2_ intensity = 15.59). These observations demonstrate for the present study that the dynamic range of the RNA-seq technology was 3,444-fold greater than that of the microarray platform.

### Correlation of observed log_2_ expression values and log_2_ fold-changes between the RNA-seq and microarray platforms

We next examined the correlation between the log_2_ expression values generated using the RNA-seq (log_2_ RPKM values) and microarray (log_2_ intensity values) platforms for all genes that passed the filtering criteria and for which a definite gene length could be determined (RPKM values cannot be computed for genes with splicing events). Spearman rank correlation coefficient (ρ) values for the 4,844 filtered genes (common to both platforms) were then calculated separately for the MAP-infected and control groups at each post-infection time point. At 2 hpi, highly significant ρ values of 0.68 (*P* < 1.0 × 10^−15^) and 0.67 (*P* < 1.0 × 10^−15^) were observed for the MAP-infected and control sample groups, respectively. Similarly, at 6 hpi, highly significant ρ values were also observed: 0.68 (*P* < 1.0 × 10^−15^) for the MAP-infected sample group and 0.66 (*P* < 1.0 × 10^−15^) for the control sample group.

Following this, log_2_ expression fold-changes were examined directly for the 5,419 genes that overlapped the RNA-seq and the microarray platform at both post-infection time points (this included the 4,844 gene transcripts detailed above, plus the 575 additional overlapping genes that exhibited alternative transcripts). Again, highly significant ρ values were observed for the correlation between log_2_ expression fold-change values for RNA-seq and the microarray platform at both 2 hpi (0.46; *P* < 1.0 × 10^−15^) and 6 hpi (0.52; *P* < 1.0 × 10^−15^).

The results of these analyses, using both log_2_ expression values and log_2_ expression fold-changes from the two post-infection time points, support the reproducibility and robustness of gene expression studies on the same samples using RNA-seq and the Affymetrix^®^ microarray platform.

## Discussion

In recent years, high-throughput functional genomics and systems analysis of the mammalian host response to a range of mycobacterial pathogens has greatly enriched scientific understanding of the immunobiology of these infections (47–51). In particular, transcriptomics and downstream systems biology analyses of the *in vitro* macrophage response to mycobacteria have been particularly informative regarding host–pathogen interactions that underlie pathogenesis and which are reflected in perturbation of host genes and cellular pathways (25, 29, 30, 32, 52–57).

Most of this research work has been performed using various types of microarray platform, which until recently, has been the technology of choice for transcriptomics studies of the host response to infection. However, during the last 6 years, RNA-seq has emerged as the most powerful tool for high-resolution interrogation of the eukaryotic transcriptome in response to external stimuli such as invasive pathogens (58–61). RNA-seq has significant advantages over microarrays for surveying the complex transcriptional landscape of multicellular organisms. For example, microarray construction and implementation requires pre-existing genome sequence information for probe design, while pre-selection of the genes and transcripts to be interrogated by the microarray may result in a biased representation of the transcriptome. In contrast, RNA-seq offers unbiased, genome-wide transcriptome profiling of host gene expression without the requirement of pre-existing genome sequence information prior to the initiation of experiments. In addition, compared to microarrays, which have a dynamic range constrained by technical factors (for example, probe saturation for highly expressed genes, or lack of detectable probe hybridization signal for lowly expressed genes), the dynamic range of RNA-seq is normally limited only by the depth of sequencing used for a particular experimental comparison, thereby leading to higher sensitivity for detection of lowly expressed transcripts. Also, where appropriate, RNA-seq data can be used to quantify alternatively spliced gene variants; identify novel transcribed genes; and study antisense transcription (28, 29, 62, 63). Consequently, for the present study, an RNA-seq approach was used to study the bovine host macrophage response to MAP infection *in vitro* across an experimental time course consisting of 2 and 6 hpi time points.

### Differential gene expression and functional biology of RNA-seq results

RNA-seq analysis demonstrated that of the 245 significantly DE genes detected at 2 hpi, 85.3% of these were upregulated in MAP-infected MDM compared to non-infected control MDM. Also at 6 hpi, 59.6% of the DE genes were upregulated in infected MDM relative to the controls (Table S2 in Supplementary Material). This pattern of a general increase in gene expression in bovine macrophages within the first 6 h of MAP infection *in vitro* has also been observed by our group and other workers (30, 64, 65). It is also noteworthy that the mean absolute log_2_ fold-change in expression for upregulated genes in MAP-infected MDM was markedly higher than for downregulated genes at both 2 hpi (1.95 versus 1.07, respectively) and 6 hpi (1.50 versus 0.92, respectively). This is consistent with results obtained by Magee and colleagues using bovine MDM infected with *M. bovis* (32).

The most upregulated DE gene (ranked by fold-change) observed at 2 hpi from RNA-seq was the *CSF3* gene (log_2_ fold-change = +8.05, Table 1), which encodes a cytokine that controls the production, differentiation, and function of granulocytes and which has also been shown to be highly upregulated in MAP-infected MDM isolated from red deer (*Cervus elaphus*) (66). It is interesting to note that Marfell and colleagues also observed that upregulation of this gene was higher in susceptible animals compared to resistant animals. The most downregulated annotated gene at 2 hpi using RNA-seq was the *RAB3A* gene (log_2_ fold-change = −2.10, Table 1), which plays an important role in intracellular vesicle and membrane trafficking (67). While this gene has not previously been shown to be associated with macrophage–mycobacteria interactions, its downregulation could reflect an aspect of the disruption of phagosome–lysosome fusion mediated by MAP to promote its survival (68).

The most upregulated DE gene (ranked by fold-change) detected at 6 hpi using RNA-seq was the *LOXL4* gene (log_2_ fold-change = +5.42, Table 2), which has not previously been associated with a functional role in macrophage–mycobacteria interactions, but has a primary role in connective tissue biogenesis (69). However, recent findings have suggested that the LOX family of proteins may also have an ancillary transcriptional regulatory function (70). The most downregulated gene at 6 hpi detected using RNA-seq was the *OPRD1* gene (log_2_ fold-change = −3.21, Table 2), which encodes an opioid receptor also not previously reported to be involved in the macrophage response to intracellular pathogens. However, it has been demonstrated that TNF-α and IL-1β can downregulate the expression of opioid receptors at the mRNA level (71).

The identification of DE genes that hitherto had no documented role in macrophage–mycobacterial interactions highlights the potential of RNA-seq for revealing novel layers of information regarding host cellular processes induced following MAP infection and the roles that these genes may play in the host immune responses to MAP infection.

Several pro-inflammatory cytokine and chemokine genes, including *CCL20*, *CXCL2*, *CXCL3*, *IL1B*, and *TNF*, were DE at the 2 hpi time point; previous studies have highlighted the important role played by the products of these genes in regulating the innate immune response to mycobacterial infection (15, 20, 23, 24). The pro-inflammatory response to infection is further supported by the perturbation of several immunological signaling pathways including CD40 signaling (Figure 4), which is required for activation of antigen-presenting cells such as the macrophage (72, 73); IL-15 signaling, which regulates pro-inflammatory cytokine production in the macrophage (74); and interferon signaling (75, 76).

Furthermore, IL-1 pro-inflammatory cytokine expression in the MAP-infected host is critical not only to protective immunity but also to MAP survival. IL-1 cytokines are key effector cytokines produced by macrophages in response to infection with MAP. Indeed, IL-1 cytokine expression was detected as early as 10 min after infection with MAP under experimental infection conditions and interestingly, co-culture systems have shown that the macrophages recruited as a result of epithelial cell-induced IL-1 cytokines can be exploited by MAP to enhance their survival within the host (77). It is noteworthy that in the present study, both *IL1A* and *IL1B* are significantly DE in MAP-infected MDM at 2 hpi (*IL1A* log_2_ fold-change = +4.9; *IL1B* log_2_ fold-change = +5.6).

In order to produce the mature forms of IL-1 cytokines, the inflammasome is required. This pro-inflammatory protein complex occurs in myeloid cells upon infection to coordinate the activation of effective anti-bacterial innate immunity (78). The exact composition of the inflammasome varies depending on the activator (e.g., bacterial toxin, bacterial components, flagellin, and dsDNA); however, it has not been well defined in bovine studies (79). Both *NLRP3* (log_2_ fold-change at 2 hpi = +2.7) and *IRAK2* (log_2_ fold-change at 2 hpi = +2.2) are important components of the NLRP3-inflammasome complex. Indeed, *Nlrp3*^−/−^ knockout mice do not produce IL-1 cytokines (80).

Other genes encoding proteins associated with induction and activation of the inflammasome include *SAA3* (2 and 6 hpi) (81) – which encodes an important acute phase protein of macrophages – and *CASP4* (6 hpi) (82) – which encodes a protease with a well-characterized role in programed cell death. In contrast, *CASP8*, which also exhibited increased expression at 6 hpi, encodes caspase 8, which has an inflammasome-blocking function (83). Therefore, *CASP8* upregulation may reflect host-directed control of inflammasome activation or, possibly, immunoevasive modulation by mycobacterial factors. Previous work has demonstrated that mycobacteria, such as *M. tuberculosis*, can block inflammasome activation as a novel immune evasion strategy (79, 84). In addition, lung infection with *M. tuberculosis* generates increased NO expression levels, which negatively regulates the NLRP3-inflammasome, thereby decreasing IL-1β production (85). Therefore, the results for MAP infection of bovine MDM may be a useful avenue for future studies regarding the interplay between bovine macrophages and MAP.

The genes encoding IL-1RN, and the anti-inflammatory cytokine IL-10 – two important regulators of IL-1 cytokine family activity – are both DE [*IL1RN* log_2_ fold-change = +1.1 (2 hpi), +1.9 (6 hpi); *IL10* log_2_ fold-change = +2.01 (2 hpi)]. Notably, IL-10 signaling is also the top ranked canonical pathway identified by IPA at 2 hpi (Figure 3). *IL10* encodes an immunosuppressive cytokine that regulates the antimicrobial activity of the macrophage, thus limiting the level of cytokine-induced tissue damage. Upregulation of IL-10 expression induced by mycobacteria has been proposed to inhibit host innate immune responses during infection resulting in enhanced pathogen survival (86–88).

Our findings support the hypothesis that the immunomodulatory mechanisms employed by MAP are reflected in the host macrophage transcriptome. Ultimately, protection against mycobacterial infection is a balance between protection and pathology (89). While there is significant activation of a pro- inflammatory immune response in MDM at 2 hpi, it is clear that this response is quickly regulated as the pro-inflammatory mediators are no longer DE at 6 hpi. In this regard, the outcome of infection is decided by the balance between pro- and anti-inflammatory mediators (24, 90–92).

### Technical comparison of RNA-seq and microarray technologies for gene expression analysis

Previously, the MDM-extracted RNA samples analyzed for the present study were examined with the pan-genomic Affymetrix^®^ GeneChip^®^ Bovine Genome Array microarray platform. Here, we have used new RNA-seq data and a re-analyzed microarray data set to perform a direct technical comparison of gene expression estimation between the two platforms. The number of DE genes identified 2 hpi was higher in the microarray compared to RNA-seq (315 versus 245), while conversely, the number of DE genes 6 hpi detected via RNA-seq analysis exceeded those detected by the microarray (574 versus 466). In total, across the two infection time points the number of DE genes was higher based on the RNA-seq data compared to the microarray data (819 versus 781; an increase of 5%). Although this increase in the number of RNA-seq-identified DE genes relative to microarray-identified DE genes is lower than that previously reported by us in a technical comparison of RNA extracted from *M. bovis*-infected and non-infected MDM, this finding is consistent with other studies demonstrating greater numbers of DE genes identified by RNA-seq compared to microarray analysis of the same samples (29, 93, 94).

The increased number of DE genes detected by RNA-seq may be attributed to the increased dynamic range of RNA-seq relative to the microarray, which permits the detection of lowly expressed DE genes between MAP-infected and non-infected control MDM (29, 94–96). Furthermore, the concordance between the two platforms, as estimated by the percentage of DE genes common to both platforms across both infection time points, was 41.36% (323/781 genes) for the microarray and 39.44% (323/819 genes) for RNA-seq. These estimates are in agreement with the concordance previously determined for a comparison of bovine MDM infected with *M. bovis* and control non-infected MDM (29). The differences observed in gene expression estimation between the two platforms may be explained by several technical and analytical factors including: (1) systematic differences in dynamic range; (2) differences in the statistical models used to analyze digital/count gene expression data such as that generated using RNA-seq and the analog/continuous data obtained from microarrays; and (3) differences in the mRNA transcripts analyzed by both platforms (for example, the probes on Affymetrix GeneChip arrays are predominantly based on sequences at the 3′ end of genes and are therefore 3′ biased, while RNA-seq read data are expected to be more equally distributed across gene transcripts) (38, 46, 97–101).

In summary, the present study describes a transcriptomics survey of the host macrophage response to MAP infection using bovine MDM as an experimental model. We have used RNA-seq data generated from MDM infected with a clinical strain of MAP across a 6 h infection time course and compared the results of the RNA-seq analysis to a comparable re-analysis of microarray data obtained using the same experimental samples. The results of this work provide new insights into macrophage-MAP interplay, highlighting potential functional roles for genes that previously have not been implicated in the host response to infection with MAP bacilli. Furthermore, the pro- and anti-inflammatory cytokines involved in the bovine MDM response to MAP infection, such as those associated with the IL-10 signaling pathway, emphasize the balance between protective host immunity and bacilli survival and proliferation. Finally, by directly comparing the performance of two transcriptomics platforms, we demonstrate that RNA-seq represents a superior technology to microarrays for *in vitro* analyses of gene expression using mammalian cells infected with intracellular bacterial pathogens.

## Conflict of Interest Statement

The authors declare that the research was conducted in the absence of any commercial or financial relationships that could be construed as a potential conflict of interest.

## Supplementary Material

The Supplementary Material for this article can be found online at http://www.frontiersin.org/Journal/10.3389/fimmu.2015.00023/abstract

## Footnotes

^1^www.bioinformatics.babraham.ac.uk/projects/fastqc

^2^http://www.ingenuity.com
