# Supplementary material for: Analysis of the Bovine Monocyte-Derived Macrophage Response to Mycobacterium avium Subspecies Paratuberculosis Infection Using RNA-seq
Source: Front Immunol. 2015 Feb 4;6:23. doi: 10.3389/fimmu.2015.00023 (PMC4316787; doi:10.3389/fimmu.2015.00023)
Supplement: Supplementary file 1 [file Presentation_1.ZIP › Supplementary Table and Figure captions.DOCX]

***Supplementary Material***

**Analysis of the bovine monocyte-derived macrophage response to *Mycobacterium avium* subspecies *paratuberculosis* infection using RNA-seq**

**Maura E. Casey^1,2^, Kieran G. Meade^2^, Nicolas C. Nalpas^1^, Maria Taraktsoglou^3^, John A. Browne^1^, Kate E. Killick^1,4^, Stephen DE. Park^1,†^, Eamonn Gormley^5^, Karsten Hokamp^6^, David A. Magee^1,‡^, David E. MacHugh^1,7^***

^1^Animal Genomics Laboratory, UCD School of Agriculture and Food Science, University College Dublin, Dublin 4, Ireland

^2^ Animal & Bioscience Research Department, Animal & Grassland Research and Innovation Centre, Teagasc, Grange, Co. Meath, Ireland

^3^Biological Agents Unit, Health and Safety Executive, Leeds, LS11 9AT, United Kingdom

^4^Systems Biology Ireland, UCD Conway Institute of Biomolecular and Biomedical Research, University College Dublin, Dublin 4, Ireland

^5^Tuberculosis Diagnostics and Immunology Research Centre, UCD School of Veterinary Medicine, University College Dublin, Ireland

^6^Smurfit Institute of Genetics, Trinity College, Dublin 2, Ireland

^7^UCD Conway Institute of Biomolecular and Biomedical Research, University College Dublin, Dublin 4, Ireland

*** Correspondence:** David E. MacHugh, Animal Genomics Laboratory, UCD Veterinary Sciences Centre, University College Dublin, Dublin 4, Ireland.

**† Present address:** IdentiGEN Ltd., Trinity Enterprise Centre, Pearse Street, Dublin 2. Ireland.

**‡ Present address:** Department of Animal Science, University of Connecticut, Storrs, CT 06269-4040, USA.

[david.machugh@ucd.ie](mailto:david.machugh@ucd.ie)

**Keywords: cattle, immune response, Johne’s disease, macrophage, microarray, *Mycobacterium avium* subspecies *paratuberculosis*, RNA-sequencing, transcriptome.**

1. **Supplementary Figures and Tables**

**Table S1:** RNA-seq library information (deconvolution, alignment and assignment of reads) for MAP-infected and control non-infected bovine MDM samples. [**Table_S1.xlsx**]

**Table S2:** Detailed information for DE genes detected using RNA-seq in MAP-infected versus control non-infected MDM samples at 2 hpi and 6 hpi. Genes with FDR-adjusted *P* values of ≤ 0.05 are highlighted. [**Table_S2.xlsx**]

**Table S3:** Biological Process GO functions identified using the GOseq package for DE genes detected using RNA-seq from MAP-infected versus non-infected control MDM at 2 hpi. [**Table_S3.xlsx**]

**Table S4:** Biological Process GO functions identified using the GOseq package for DE genes detected using RNA-seq from MAP-infected versus non-infected control MDM at 6 hpi. [**Table_S4.xlsx**]

**Table S5:** Canonical pathways identified using IPA for RNA-seq DE gene results from MAP-infected and non-infected control MDM at 2 hpi. [**Table_S5.xlsx**]

**Table S6:** Canonical pathways identified using IPA for RNA-seq DE gene results from MAP-infected and non-infected control MDM at 6 hpi. [**Table_S6.xlsx**]

**Table S7:** Detailed information for DE genes detected using the Affymetrix^®^ bovine microarray in MAP-infected versus control non-infected MDM samples at 2 hpi and 6 hpi. Genes with FDR-adjusted *P* values of ≤ 0.05 are highlighted. [**Table_S7.xlsx**]

**Figure S1:** Analysis steps for primary RNA-seq data generated from MAP-infected and control MDM samples. A) Pie chart showing results from quality-filtering and sequencing adaptor removal. B) Pie chart showing results of sequence read alignment to the bovine genome. C) Pie chart showing assignment of sequence reads to annotated bovine genes. [**Figure S1.tif**]

**Figure S2:** MDS plots of RNA-seq and microarray gene expression data from seven MAP-infected MDM samples and seven control MDM samples at 2 hpi and 6 hpi. A) 2 hpi RNA-seq MDS plot generated from 11,813 genes that passed all data filtering prior to differential gene expression analysis. B) 6 hpi RNA-seq MDS plot generated from 11,813 genes that passed all data filtering prior to differential gene expression analysis. C) 2 hpi microarray MDS plot generated from 11,259 informative probes that passed all data filtering prior to differential gene expression analysis. D) 6 hpi microarray MDS plot generated from 11,259 informative probes that passed all data filtering prior to differential gene expression analysis. Red circles indicate MAP-infected MDM samples; Green circles indicate control non-infected MDM samples. [**Figure S2.tif**]

**Figure S3:** Venn diagrams showing numbers of DE genes identified from RNA-seq and microarray data. A) DE genes detected in MAP-infected MDM versus control non-infected MDM using RNA-seq at 2 hpi. B) DE genes detected in MAP-infected MDM versus control non-infected MDM using RNA-seq at 6 hpi. Sets of upregulated genes are represented in red and sets of downregulated genes are shown in green. [**Figure S3.tif**]
