# Supplementary figures and images for: Analysis of the Bovine Monocyte-Derived Macrophage Response to Mycobacterium avium Subspecies Paratuberculosis Infection Using RNA-seq
Source: Front Immunol. 2015 Feb 4;6:23. doi: 10.3389/fimmu.2015.00023 (PMC4316787; doi:10.3389/fimmu.2015.00023)

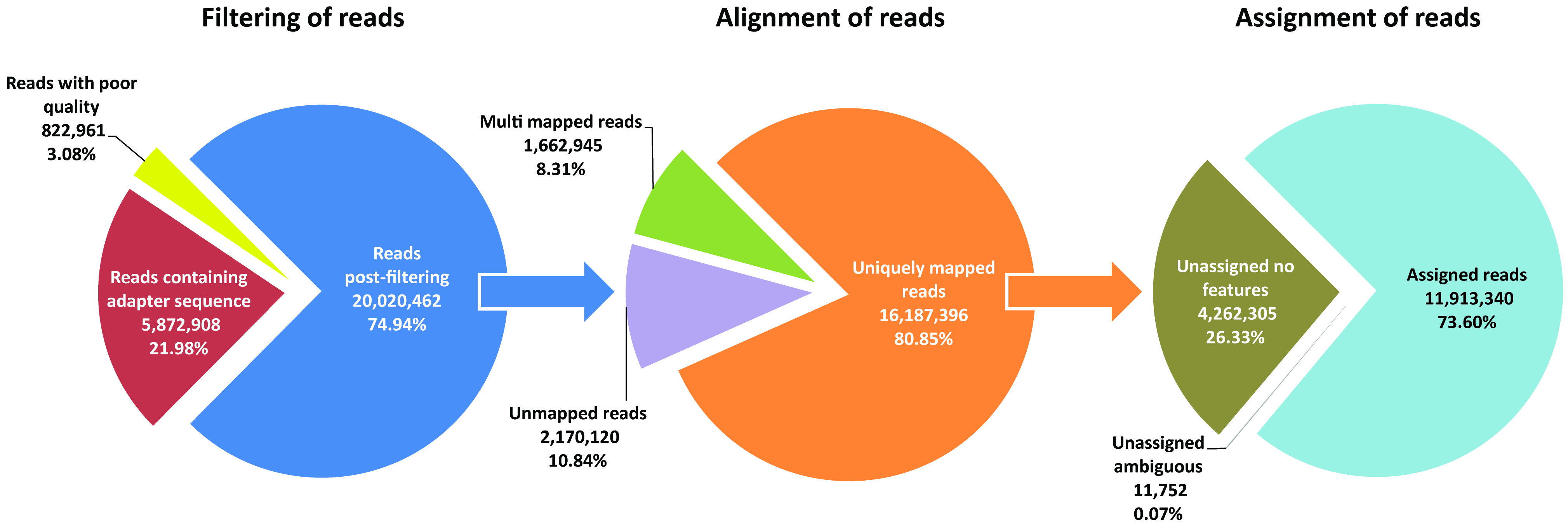

Supplement: Supplementary file 1 [file Presentation_1.ZIP › Figure S1.TIF]

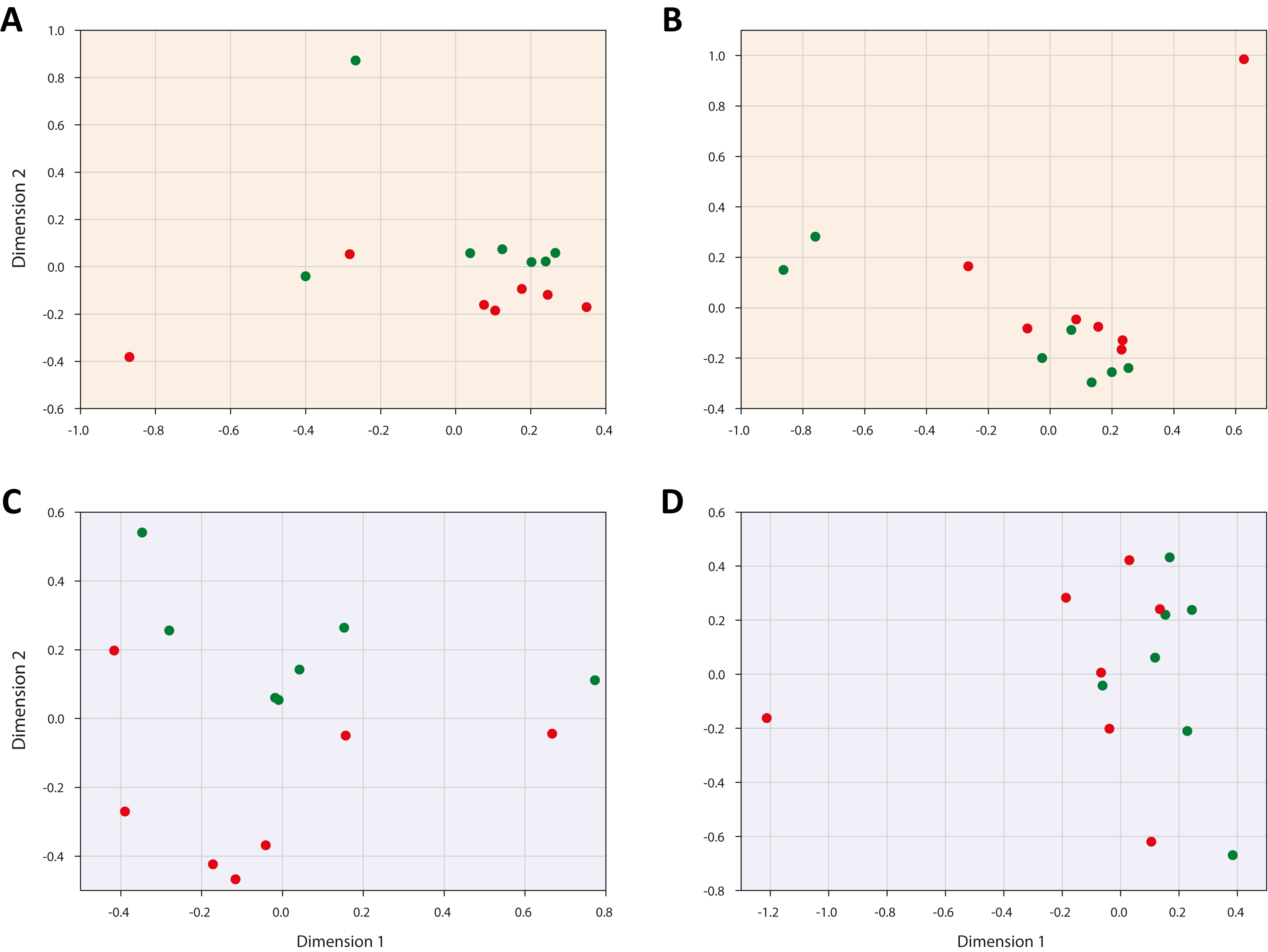

Supplement: Supplementary file 1 [file Presentation_1.ZIP › Figure S2.TIF]

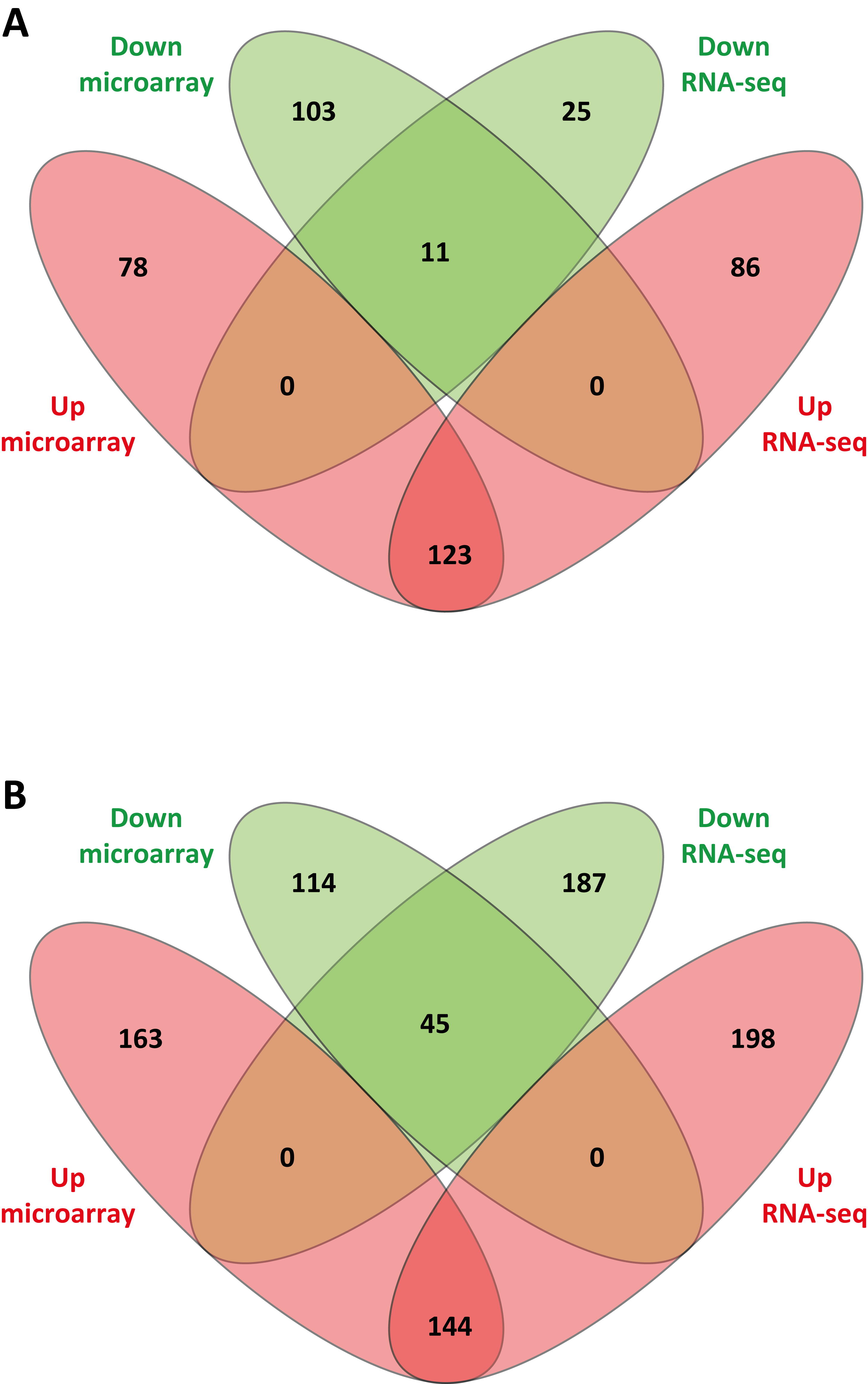

Supplement: Supplementary file 1 [file Presentation_1.ZIP › Figure S3.TIF]
